# Characterization of an Artificial Liver Support System-Related Vasovagal Reaction

**DOI:** 10.1155/2020/6313480

**Published:** 2020-07-13

**Authors:** Shanshan Ma, Zhongyang Xie, Huafen Zhang, Jiangcheng Rong, Lingjian Zhang, Ya Yang, He Jiang, Xiaowei Xu, Lanjuan Li

**Affiliations:** ^1^State Key Laboratory for Diagnosis and Treatment of Infectious Diseases, The First Affiliated Hospital, College of Medicine, Zhejiang University, Hangzhou, China; ^2^National Clinical Research Center for Infectious Diseases, The First Affiliated Hospital, College of Medicine, Zhejiang University, Hangzhou, China; ^3^Collaborative Innovation Center for Diagnosis and Treatment of Infectious Diseases, The First Affiliated Hospital, College of Medicine, Zhejiang University, Hangzhou, China; ^4^Department of Infectious Diseases, The First Affiliated Hospital, College of Medicine, Zhejiang University, Hangzhou, China

## Abstract

**Objective:**

An artificial liver support system (ALSS) is an effective therapy for patients with severe liver injury. A vasovagal reaction (VVR) is a common complication in various treatment settings but has not been reported previously in ALSS.

**Methods:**

This study retrospectively evaluated patients who suffered an ALSS-related VRR between January 2018 and June 2019. We collected data from VVR episodes including onset time, duration, changes in heart rate (HR) and blood pressure (BP), and drug treatment.

**Results:**

Among 637 patients who underwent ALSS treatment, 18 were included in the study. The incidence of VVR was approximately 2.82%. These patients were characterized by a rapid decrease in BP or HR with associated symptoms such as chest distress, nausea, and vomiting. The majority of patients (78%) suffered a VVR during their first ALSS treatment. Sixteen patients (89%) had associated symptoms after treatment began. Sixteen patients (89%) received human albumin or Ringer's solution. Atropine was used in 11 patients (61%). The symptoms were relieved within 20 min in 15 patients and over 20 min in 3 patients.

**Conclusions:**

A VVR is a rare complication in patients with severe liver injury undergoing ALSS treatment. Low BP and HR are the main characteristics of a VVR.

## 1. Introduction

Liver failure is a severe clinical syndrome characterized by hepatic encephalopathy, jaundice, coagulopathy, and a high mortality rate. Hepatitis viruses, drugs, or toxins can precipitate acute liver failure in patients with or without chronic liver disease [1]. Unfortunately, liver transplantation currently remains the only definitive treatment option for irreversible liver failure [2]. Approximately 90% of patients are cured after liver transplantation [3, 4], but there is a serious shortfall of donors, especially in China, and costs are considerable for many families. Some patients recover spontaneously with standard medical therapy.

Artificial liver support systems (ALSSs) are a bridge to liver transplantation or recovery after liver failure and are useful means of improving biochemical parameters and clinical symptoms [5]. An ALSS is an external support system that can remove several types of harmful substances while supplementing essential substances, thereby improving the internal environment and create conditions for the regeneration of liver cells and restoration of liver function. There are various modalities connecting treatment units either in series or in parallel such as plasma exchange (PE) combined with hemofiltration (HF), molecular absorbent recirculatory systems, fractionated plasma separation and absorption (using the Prometheus system), and double plasma molecular absorption systems (DPMAS).

The safety of most ALSS devices has been well established by clinical studies [2]. Commonly reported side effects of ALSSs include filter clotting, leukocytosis, hypotension, bleeding, anaphylaxis, and thrombocytopenia [2]. A vasovagal reaction (VVR) is an abnormal autonomic imbalance precipitated by pain, hunger, heat, imagined or real exposure to bodily harm, certain medical and surgical procedures, cardiac spasm, distention of viscera, and pleural or peritoneal irritation, which is characterized by hypotension followed by paradoxical bradycardia [6]. More seriously, strong vagal stimulation may lead to arrhythmias, syncope, transient asystole, seizures, or death [7–10]. To the best of our knowledge, a VVR during ALSS treatment has not been reported previously. Here, we describe 18 patients who experienced a VVR during treatment with an ALSS.

## 2. Material and Methods

### 2.1. Patients

We retrospectively reviewed the medical records of patients who underwent ALSS treatment for liver failure at our hospital and suffered an ALSS-related VVR from January 1, 2018 to June 30, 2019.

A VVR was defined as the presence of at least one of the following criteria with associated symptoms: (1) bradycardic episode (heart rate (HR) < 60 beats/min (bpm)) and hypotension (systolic blood pressure (SBP) < 100 mmHg) in the supine position or (2) development of syncope [6, 11]. Common symptoms include pallor, faintness, dizziness, sweating, nausea, shivering, vomiting, heat intolerance, cold intolerance, and loss of consciousness [12]. Exclusion criteria were heart failure, myocardial infarction, or other severe liver diseases. A VVR was noted only if the reaction occurred following the entire procedure, including femoral vein catheter placement.

### 2.2. Data Collection

Data collected at the time of ALSS treatment included age, sex, body mass index, etiology of liver failure, serum total bilirubin (TB) level, alanine and aspartate aminotransferase (ALT, AST) levels, glucose level, and international normalized ratio (INR). The diastolic blood pressure (DBP), SBP, and HR after extracorporeal circulation were used as the baseline data. When a VVR occurred, the onset time, minimum BP and HR, symptoms, treatment measures, and recovery time were all recorded in detail.

### 2.3. ALSS Treatment

The indications for ALSS treatment were based on the Chinese Guideline for Diagnosis and Treatment of Liver Failure [13] and include (1) early- or intermediate-stage acute, subacute, and acute-on-chronic liver failure (prothrombin activity of 20–40%); (2) end-stage liver disease awaiting liver transplantation; (3) rejection after liver transplantation or a nonfunctional transplanted liver; and (4) severe cholestasis (TB > 10 mg/dL) due to ineffective medical treatment. PE combined with HF was administered to five patients. PE was performed for 2 h each time using the EC-40 W plasma separator (Asahi Kasei, Japan), and the replacement fluid contained 500–1,000 mL albumin plus 1,500–2,000 mL fresh frozen plasma, for a total exchange volume of 2,500–3,000 mL. Subsequently, HF was performed continuously for approximately 4–6 h using the BLS816G (SORIN Group, Italy) at a filtrate flow rate of 50 mL/kg/h. The entire procedure lasted 6–8 h. A DPMAS was used to treat 13 patients. The plasma was separated using the EC-40 W device (Asahi Kasei), purified by an anion exchange resin column (HA330-II, Jian Fan, China) and a bilirubin adsorption column (BS330, Jian Fan), and finally returned to the patient. ALSS treatment was administered using the IQ21 machine (Asahi Kasei). A double-lumen catheter was inserted into the femoral vein to obtain vascular access. Heparin was given to prevent coagulation, with the dosage adjusted based on the transmembrane pressure and activated partial thromboplastin time. A plasma allergy was prevented by administering 5 mg dexamethasone before ALSS treatment.

### 2.4. Statistical Analysis

Data were compared using Student's *t*-test conducted in Prism, version 8.0.0 (GraphPad, San Diego, CA). Statistical significance was defined at *P* < 0.05.

## 3. Results

Among 637 patients who underwent ALSS treatment, a VVR occurred in 18 (2.82%), who were included in the study. The clinical features of the 18 patients (9 males and 9 females) are shown in Table 1. The mean age of the patients was 53.2 (range 22–74) years, and the mean body mass index was 21 (range 18–28) kg/m^2^; excluding patient 17 who was too weak to obtain height or weight data. The most common etiology of liver failure was hepatitis B virus (56%), followed by drugs (22%). The glucose levels in all patients were normal to high (range 6.7–16.8 mmol/L) at the time of the VVR. All patients exhibited increased aminotransferase levels: the ALT level ranged from 39 to 427 (mean 138) U/L and the AST level from 34 to 383 (mean 129) U/L. Jaundice was present in all 18 patients. The serum TB level ranged from 175 to 583 (mean 358) *μ*mol/L. In five patients, coagulation dysfunction was present, with an INR level ≥ 1.5 (the peak INR level was 2.78 in patient 15).

**Figure 1:**
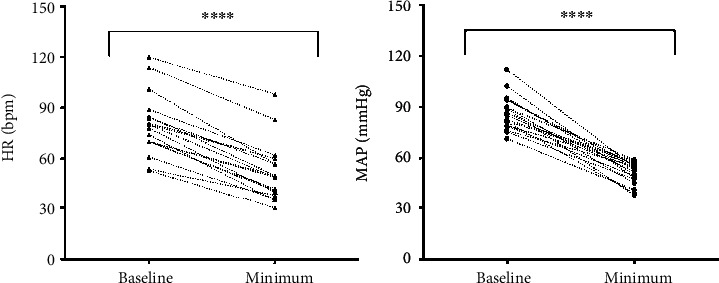
A plot of the individual HR (a) and MAP (b) at baseline and minimum during VVR. Both HR and MAP decreased significantly between two periods of time. VVR: vasovagal reaction; MAP: mean blood pressure; HR: heart rate. ^∗∗∗∗^*P* < 0.0001.

Detailed information on ALSS treatment and the VRRs, including modality of ALSS, VVR duration, changes in HR and BP, and treatment provided for the VVR, are listed in Table 2. Of the 18 patients, 13 (72%) were managed using a DPMAS, and the remaining 5 (28%) received PE combined with HF. The majority of patients (78%) suffered a VVR during their first ALSS treatment. Sixteen patients experienced the relevant symptoms after treatment commenced and the onset time of VVR ranged from 10 to 100 min. Only two patients experienced symptoms during femoral vein catheter placement. The VVR was associated with significant changes in BP and HR. The mean basal and minimum HR were 80 and 52 bpm, respectively, and the mean basal and minimum SBP were 119 and 72 mmHg, respectively. Trends in HR and mean arterial pressure are described in Figure 1. Dizziness, sweating, nausea, and chest distress were frequently reported by these patients.

**Figure 2:**
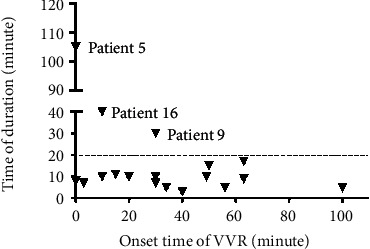
A plot of individual onset time and time of duration of VVR. VVR: vasovagal reaction.

During treatment, most patients (89%) received human albumin or Ringer's solution to increase the circulating blood volume. Atropine, as an effective drug for blocking a VVR, was used in more than half of the patients (61%). Six patients received dopamine with or without aramine. The VVR duration was short (<20 min) in 15 patients and more than 20 min in 3 patients, ranging from 3 to 105 min. The VVR time of onset and duration in all 18 patients are shown in Figure 2.

## 4. Discussion

We detected an incidence of a VVR occurring during ALSS treatment of 2.82%. There are no previously published data on VVR incidence for comparison with our results. It is important differentiate VVR from hypoglycemia. VVR is a rare complication of ALSS treatment and is more frequently associated with spine procedures [14], blood donation [15], cerebral angiography via femoral catheterization [11], and percutaneous coronary intervention [16]. In the general blood donor population, the VVR rate was reported to be 1.4%. VVR during spinal injection was found to be a common adverse event, with a rate of 0–8.6. [14] As for PE treatments, it was reported that the VVR rate was about 0.5%, in which the patients were diagnosed with the Guillain-Barre syndrome, myasthenia gravis, and so on, but liver injury were not included [17]. Compared with other conditions, a VVR incidence of 2.82% may be considered moderate.

In the present study, all patients during the VVR experienced a remarkable decrease in BP or HR accompanied by symptoms such as dizziness and sweating. Symptoms and signs returned to normal within 3–105 min, which is similar to the duration (from several minutes to 2 h) reported in a study of VVR associated with cerebral angiography [11]. Sixteen (89%) instances of VVR occurred during normal ALSS treatment, ranging from 10 to 100 min after treatment commenced. The remaining two instances of VVR occurred during femoral vein catheter placement. In a study on VVR occurring during blood donation [18], 54.4% of VVR episodes occurred during blood collection, 28.6% after collection and 9.8% after entering the refreshment area; 1.4% occurred even later, after the donor had left the site. In addition, 5.9% of VVR incidents was associated with blood test procedures requiring venipuncture. In short, VVR occurred before, during, and after blood collection, whereas ALSS-related VVR occurred mainly before and during ALSS treatment.

The vagus nerve runs throughout the vascular endothelial system. Physiologically, a VVR is believed to be caused by the initial increase in BP and HR due to an adrenergic response, followed by overcompensation by a sudden withdrawal of sympathetic tone and increased parasympathetic tone, resulting in splanchnic vasodilation, bradycardia, and decreased cerebral blood flow [19]. Hypermetabolism in the adrenal gland responsible for synthesizing and secreting catecholamines is indicated by increased adrenal fluorodeoxyglucose accumulation on positron emission tomography/computed tomography [20]. Elements such as fear, pain, and a bad mood can be triggers, leading to a strong autonomic response [12]. Internal factors such as hyperventilation, hypotension, and stress may also precipitate a VVR in some cases [21]. Alcohol ingestion may predispose individuals to a VVR in the clinical setting [22].

Patients require femoral vein catheter placement before ALSS treatment. Two patients experienced a VVR during this procedure. As reported by other studies, patients who received a vascular interventional examination or therapy were more likely to develop a VVR, which was commonly seen at the time of sheath placement [11]. A poor response to intraoperative local anesthesia, which may increase the patient's pain sensitivity, and stimulation of the vascular intima by a guide wire or catheter both can cause enhanced excitability of the vascular vagus nerve. On the other hand, patient anxiety or even insomnia may stimulate the pressure transducers in the left ventricle and carotid artery, leading to enhanced vagus nerve excitability [11]. When treatment begins, a portion of the blood flows into the pipeline, which results in relative hypovolemia, similar in some respects to blood donation. In patients with liver failure, toxins typically accumulate in the blood vessels to cause an attenuated vasoconstrictor response. As a result, mobilization of the peripheral venous blood and net fluid absorption from tissues to blood decrease [23].

VVR is an unexpected high-risk condition that may cause irreversible harm including arrhythmias, syncope, myocardial infarction, transient asystole, and seizures even death to patients without timely treatment [7–10, 24]. Most patients who develop VVR could recover after timely and accurate treatment in previous study [6], the same as we have observed. In our 18 patients, therapy for symptomatic hypotension and bradycardia was administered according to the judgement of the clinician. The treatment measures included stopping the pump of the machine, change in position (supine position with the head turned to one side or the Trendelenburg position), and fluid infusion. The final measure was intravenous injection of atropine, dopamine, aramine, or adrenaline. All patients recovered after timely and appropriate treatment. According to our experience and a literature review, we devised a suggested treatment algorithm. First, operation of the ALSS device should be stopped to reduce stimulation and prevent a further reduction of the circulating blood volume. The patient's anxiety should be assuaged by reassurance and “talk-esthesia.” [12] Second, reclining the patient rapidly into the Trendelenburg position helps facilitate perfusion to the brain [25]. Hand holding will help increase peripheral resistance and venous return [26]. Third, sufficient fluid infusion is essential. Human albumin and Ringer's solution are optional. If these measures are ineffective, 0.5 mg atropine should be injected intravenously at an appropriate dosage to reverse vagal cardiac stimulation. If the BP drops markedly, dopamine and aramine should be used. If asystole occurs, the emergency activation system should be initiated.

In an ALSS treatment setting, these steps can be taken to minimize the risk of a VVR as much as possible. It has been reported that during blood donation, the fear of drawing blood was associated with an approximately threefold increase in the odds of experiencing a VVR [27]. Thus, humanistic concern and psychological counseling are beneficial. Before treatment, clinicians should explain the entire ALSS procedure and related complications to the patient in detail to eliminate their fear and anxiety. The support of a family member or trusted friend is integral [12]. Patients should be encouraged to eat regularly and drink adequate fluids while avoiding alcohol before treatment [12]. At the time of femoral vein catheter placement, sufficient local anesthesia may reduce pain stimulation [16]. During ALSS treatment, medical personnel can communicate with the patient to create a nonthreatening and relaxing environment. Furthermore, the BP, HR, and subjective symptoms of the patient should be closely monitored, since early discovery and treatment of VVR can prevent severe outcomes.

The present study has some limitations. In this retrospective analysis, the data were collected in a strict prospective manner utilizing electronic medical records. The number of cases was limited. Furthermore, data following catheter removal were unavailable. A larger-scale prospective study is required to validate our findings.

## 5. Conclusions

To conclude, a VVR is a rare complication that can occur before or during ALSS treatment for liver failure, including during catheter placement. Clinicians should be aware of the possibility of a VVR and take the appropriate measures to prevent its occurrence.

## Figures and Tables

**Table 1:** Clinical characteristics of 18 cases of ALSS-associated VVR.

Variable	Age (years)	Sex	BMI (kg/m^2^)	Etiology of liver injury	Glu (mmol/L)	ALT (U/L)	AST (U/L)	TBil (*μ*mol/L)	INR
Patient 1	49	Male	23	Hepatitis B virus	9.7	57	135	583	2.15
Patient 2	62	Female	24	Hepatitis B virus	13.4	53	69	529	1.47
Patient 3	71	Male	21	Mixed	16.8	427	243	283	2.71
Patient 4	50	Female	19	Drugs	8.1	79	74	337	0.96
Patient 5	53	Male	21	Hepatitis B virus	7.8	183	74	202	0.92
Patient 6	59	Female	19	Cholestasis	10.2	425	383	366	1.19
Patient 7	73	Female	18	Drugs	14.9	213	249	175	0.92
Patient 8	54	Male	21	Hepatitis B virus	14.2	91	93	332	1.48
Patient 9	59	Female	21	Drugs	8.9	124	214	254	1.64
Patient 10	69	Female	22	Drugs	9.3	54	34	353	0.97
Patient 11	27	Male	19	Hepatitis B virus	8.7	97	89	372	1.41
Patient 12	62	Female	18	Cholestasis	12.4	151	69	333	1.12
Patient 13	44	Male	21	Hepatitis B virus	9.7	39	58	322	1.16
Patient 14	22	Male	23	Hepatitis B virus	6.7	77	94	401	1.3
Patient 15	74	Female	21	Hepatitis B virus	10.8	60	59	322	2.78
Patient 16	26	Male	28	Hepatitis B virus	9	113	165	548	1.49
Patient 17	67	Female	Unavailable	Mixed	11.6	92	133	398	2.68
Patient 18	37	Male	26	Hepatitis B virus	8.5	146	82	341	1.9

Note. ALSS: artificial liver support system; VVR: vasovagal reaction; BMI: body mass index; ALT: alanine aminotransferase; AST: aspartate aminotransferase; TBil: total bilirubin; INR: international normalized ratio; Glu: glucose.

**Table 2:** Detailed procedural features of ALSS and VVR.

					Baseline			Minimum				
	Modality of ALSS	Treatment times of ALSS	Onset time of VVR (since the treatment began)	Time of duration	HR (bpm)	SBP (mmHg)	DBP (mmHg)	HR (bpm)	SBP (mmHg)	DBP (mmHg)	Symptoms	Drug treatment
P1	PE+CRRT	1	30 minutes	7 minutes	81	100	64	60	78	44	Chest distress, waist discomfort	Dopamine 0.8 mg, aramine 0.4 mg, Ringer's solution
P2	DPMAS	1	30 minutes	10 minutes	53	114	61	30	61	44	Chest distress, nausea, dizziness, yawning	Adrenaline 0.04 mg, human albumin, Ringer's solution
P3	PE+CRRT	1	31 minutes	7 minutes	101	156	90	57	74	45	Yawning, waist discomfort, defecation	Human albumin
P4	DPMAS	3	63 minutes	17 minutes	70	108	70	41	85	45	Abdominal distension	Atropine 0.5 mg, dopamine 3 mg, Ringer's solution
P5	DPMAS	1	During catheter placement, 20 minutes	5 minutes, 70 minutes	78	109	64	40	76	47	Nervousness	Atropine 0.25 mg, Ringer's solution
P6	DPMAS	1	49 minutes	10 minutes	70	102	56	49	58	33	Palpitation, sweating	Atropine 0.5 mg, Ringer's solution
P7	DPMAS	1	20 minutes	10 minutes	85	109	67	50	56	31	Chest distress, nausea, dizziness	Dopamine 20 mg, human albumin
P8	DPMAS	1	34 minutes	5 minutes	61	124	79	36	79	42	Chest distress	Atropine 0.5 mg
P9	DPMAS	1	30 minutes	30 minutes	74	120	68	35	67	45	Chest distress, nausea, dizziness	Atropine 0.5 mg, dopamine 20 mg, Ringer's solution
P10	DPMAS	1	40 minutes	3 minutes	70	132	68	42	52	31	Chest distress	Atropine 0.5 mg, Ringer's solution
P11	DPMAS	3	56 minutes	5 minutes	80	120	70	49	83	39	Abdominal pain	Atropine 0.5 mg, Ringer's solution
P12	DPMAS	2	100 minutes	5 minutes	54	142	72	38	72	40	Urination, abdominal distension	Atropine 0.5 mg, Ringer's solution, dopamine 20 mg, human albumin
P13	DPMAS	1	63 minutes	9 minutes	89	119	82	62	79	44	Abdominal discomfort, nausea	Human albumin
P14	DPMAS	1	50 minutes	15 minutes	80	107	69	60	89	42	Dizziness, tinnitus	Ringer's solution
P15	PE+CRRT	3	10 minutes	10 minutes	70	125	72	50	79	49	Sore back	Human albumin, Ringer's solution
P16	DPMAS	1	10 minutes	40 minutes	114	114	78	83	63	36	Chest distress, palpitation, nausea, vomiting	Dexamethasone 3 mg, atropine 0.5 mg, dopamine 6 mg, aramine 3 mg, Ringer's solution
P17	PE+CRRT	1	15 minutes	11 minutes	120	112	57	98	67	38	Yawning, dry mouth	Atropine 0.25 mg
P18	Li-ALS	1	During catheter placement	8 minutes	84	135	86	57	75	35	Nervousness, chest distress, dizziness	Atropine 0.5 mg, Ringer's solution

Note. ALSS: artificial liver support system; VVR: vasovagal reaction; PE: plasma exchange; CRRT: continuous renal replacement therapy; DPMAS: double plasma molecular absorb system; Li-ALS: Li's artificial liver system; HR: heart rate; SBP: systolic blood pressure; DBP: diastolic blood pressure.

## Data Availability

The data used to support the findings of this study are included within the article.
